# Challenges and Limitations of Sequential MET ‐ TKI Therapy in METex14 ‐ Positive NSCLC With a Focus on Non‐ ILD Toxicities: A Case Report

**DOI:** 10.1002/cnr2.70458

**Published:** 2026-01-16

**Authors:** Akina Nigi, Toshikazu Kumasa, Keisuke Iwamoto, Hidetoshi Itani, Shigeto Kondou, Junji Uraki

**Affiliations:** ^1^ Department of Respiratory Medicine Japanese Red Cross Ise Hospital Ise Mie Japan; ^2^ Department of Radiology Japanese Red Cross Ise Hospital Ise Mie Japan

**Keywords:** immune checkpoint inhibitor, interstitial lung disease, MET exon 14 skipping mutation, MET‐TKI rechallenge, nonsmall cell lung cancer

## Abstract

**Background:**

Nonsmall cell lung cancer (NSCLC) with mesenchymal‐epithelial transition exon 14 skipping mutation (METex14) represents a distinct molecular subtype with limited therapeutic options. Selective MET tyrosine kinase inhibitors (MET‐TKIs) such as tepotinib, capmatinib, and gumarontinib have improved outcomes, but toxicities frequently limit their use. Previous case reports have described sequential rechallenge with two MET‐TKIs and, in rare cases, with three agents including an investigational drug. To our knowledge, this is the first reported case worldwide of sequential treatment with all three MET‐TKIs currently approved in Japan—tepotinib, capmatinib, and gumarontinib.

**Case:**

We report a 72‐year‐old man with METex14‐positive NSCLC who underwent surgery and adjuvant chemotherapy, later developing pleural dissemination. Tepotinib was discontinued after 2 months due to Grade 3 interstitial lung disease (ILD). Following chemotherapy and immune checkpoint inhibitors, capmatinib was introduced but stopped within 10 days for fever, mucositis, and possible ILD. Gumarontinib was subsequently initiated, but treatment was interrupted on Day 36 due to Grade 2 hepatotoxicity before resumption at a reduced dose.

**Conclusions:**

Although all three MET‐TKIs share similar mechanisms of action, they exhibit differing toxicity profiles. In this case, treatment discontinuation was not due to ILD recurrence but rather to distinct non‐ILD adverse events. Furthermore, the toxicities experienced by the patient varied between agents, suggesting that rechallenge may remain a viable strategy depending on individual tolerance. Careful toxicity monitoring and personalized risk assessment are essential when considering sequential MET‐TKI therapy.

**Abbreviations**
ALT: alanine aminotransferase
AST: aspartate aminotransferase
Bev: bevacizumab
CBDCA: carboplatin
CT: computed tomography
CTCAE: Common Terminology Criteria for Adverse Events
CYP3A4: cytochrome P450 3A4
DTX: docetaxel
ICI: immune checkpoint inhibitor
ILD: interstitial lung disease
MET: mesenchymal‐epithelial transition
NSCLC: nonsmall cell lung cancer
PD‐L1: programmed death‐ligand 1
Pem: pemetrexed
PET‐CT: positron emission tomography–computed tomography
Ram: ramucirumab
TKI: tyrosine kinase inhibitor
TMB: tumor mutation burden
UFT: tegafur–uracil
UGT1A1: uridine 5′‐diphospho‐glucuronosyltransferase 1A1

## 1 Introduction

Nonsmall cell lung cancer (NSCLC) harboring mesenchymal‐epithelial transition exon 14 skipping mutations (METex14) represents a distinct molecular subtype, comprising approximately 3% of all NSCLC cases. Without targeted therapy, this subtype is associated with poor prognosis. Use of selective MET tyrosine kinase inhibitors (TKIs) such as tepotinib and capmatinib has significantly improved patient outcomes. In pivotal trials, the median overall survival was 17.1 months with tepotinib and 21.5 months with capmatinib [1, 2].

Although MET‐TKI‐induced interstitial lung disease (ILD) is uncommon, with an incidence of 3%–6% in clinical trials, it can be severe enough to negatively limit treatment. Other adverse events, including rash, edema, and hepatotoxicity, are more common and may also lead to discontinuation.

Several case reports have described successful rechallenge with different MET‐TKIs after resolution of ILD [3, 4, 5, 6]. However, data on sequential MET‐TKI treatment following multiple toxicities are limited. The effects of chemotherapy and immune checkpoint inhibitors (ICIs) on subsequent MET‐TKI are unclear. There are reports that MET‐TKI after ICI increases adverse events.

Herein, we present the case of a patient with METex14‐positive NSCLC who sequentially received tepotinib, capmatinib, and gumarontinib, each associated with distinct toxicities, leading to treatment discontinuation. By comparing four previously reported cases, we argue that non‐ILD toxicities may pose a more significant barrier to durable rechallenge than previously recognized.

## 2 Case Presentation

**FIGURE 1:**
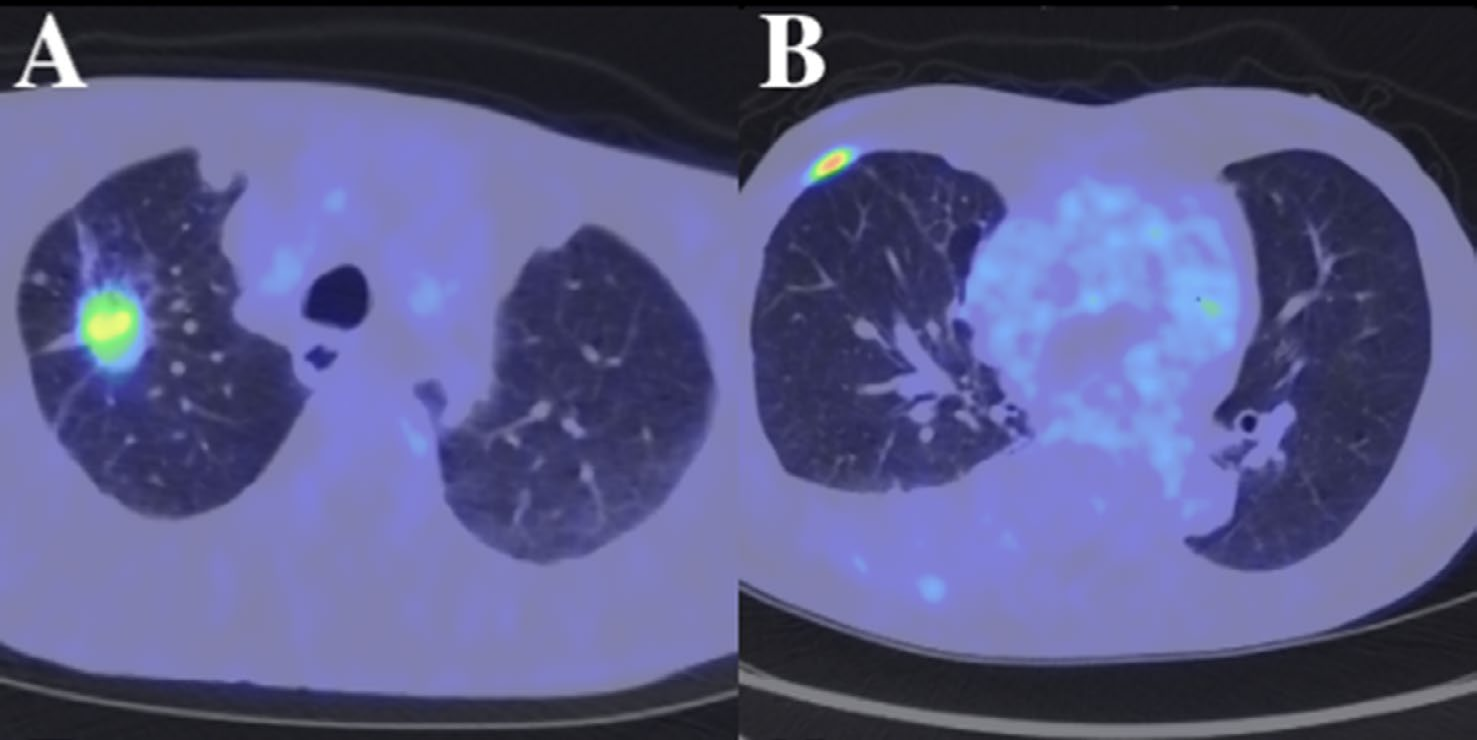
Initial diagnosis and disease recurrence. (A) Base line PET‐CT imaging prior to initial surgery demonstrating a fluorodeoxyglucose (FDG)‐avid lesion in the right upper lobe, consistent with primary lung adenocarcinoma. (B) Follow‐up chest CT approximately 22 months postoperatively, showing pleural dissemination suggestive of disease recurrence.

In March 2022, a 72‐year‐old Japanese man was diagnosed and treated at the Japanese Red Cross Ise Hospital (Mie, Japan). He had a history of atrial fibrillation and hypertension, both diagnosed over 5 years prior and well‐controlled with medication. The patient underwent a right upper lobectomy with systematic mediastinal and hilar lymph node dissection, including stations 2R, 4R, 10R, 11R, and 12R, for stage IIA (pT2aN0M0) lung adenocarcinoma (Figure 1A).

**FIGURE 2:**
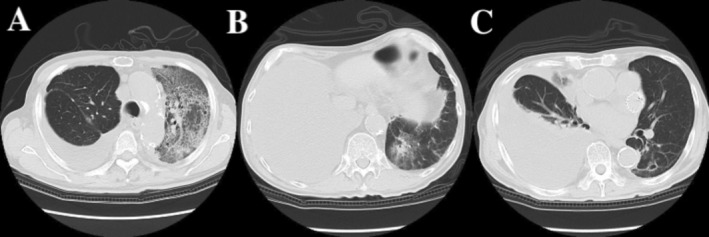
Imaging of adverse events during sequential MET‐TKI therapy. (A) Diffuse ground‐glass opacities in the left lung observed on CT after 2 months of tepotinib therapy, consistent with Grade 3 drug‐induced ILD. (B) Newly developed ground‐glass opacities after 10 days of capmatinib therapy, raising suspicion of ILD (Grade 1). (C) Chest CT on Day 36 of gumarontinib therapy. No ILD findings were observed, although right pleural effusion was present. Gumarontinib was discontinued due to Grade 2 hepatotoxicity.

Postoperative molecular profiling in April 2022 revealed a MET exon 14 skipping mutation, with EGFR, ALK, and ROS1 mutations negative. The PD‐L1 tumor proportion score was 10%. The patient received oral tegafur‐uracil (UFT) as adjuvant chemotherapy, planned for 2 years. In January 2024, routine imaging revealed pleural dissemination (Figure 1B). UFT was discontinued, and tepotinib (500 mg/day) was initiated. Two months after starting tepotinib, in March 2024, the patient developed fatigue and dyspnea. Chest CT showed diffuse ground‐glass opacities in the left lung (Figure 2A), and he was diagnosed with Grade 3 ILD. Bronchoscopy with bronchoalveolar lavage fluid was not performed because the patient's respiratory condition required urgent initiation of corticosteroid therapy. The diagnosis was therefore made based on clinical presentation and radiological findings.

Treatment was initiated with steroid pulse therapy (methylprednisolone 1000 mg/day for 3 days), followed by oral prednisolone (5 mg/day) and tacrolimus (0.5 mg/day). Resolution of CT findings and symptom improvement confirmed treatment efficacy. After ILD recovery in May 2024, confirmed by imaging and clinical improvement, the patient underwent two cycles of carboplatin plus pemetrexed, followed by two cycles of pembrolizumab in combination, and then four cycles of pembrolizumab monotherapy. However, in October 2024, increased pleural effusion and dyspnea were noted, and disease progression was assessed. In November 2024, capmatinib was started, but discontinued on Day 10 due to high‐grade fever (39°C), oral ulcers, and new ground‐glass opacities (Figure 2B), raising suspicion of Grade 1 possible ILD. This was followed by six cycles of docetaxel (DTX) and ramucirumab (Ram) between December 2024 and April 2025, which resulted in temporary disease control. Subsequently, due to recurrent pleural effusion, gumarontinib (300 mg/day) was initiated in May 2025. On Day 36 of gumarontinib treatment (June 2025), the patient experienced fatigue, and laboratory tests revealed Grade 2 hepatotoxicity (AST 92 IU/L, ALT 92 IU/L), leading to temporary discontinuation. Although right pleural effusion was present, no evidence of ILD was observed on CT (Figure 2C). After recovery of liver function, treatment was resumed at a reduced dose of 150 mg/day. No hepatic dysfunction was observed on testing 2 weeks after resumption. After an additional 57 days of treatment, in August 2025, gumarontinib was discontinued due to renal dysfunction and edema. Subsequently, the patient was maintained on carboplatin plus pemetrexed and bevacizumab, which is ongoing at the time of this report. The clinical course is outlined in Table 1 for clarity. The severity of events was graded according to the Common Terminology Criteria for Adverse Events (CTCAE), version 5.0.

**TABLE 1:** Timeline of the patient's clinical course showing treatments, adverse events, and reasons for therapy changes.

Date	Event/treatment	Notes/duration
2022 early February	Right upper lobectomy + lymph node dissection	StageIIA No LN metastasis
2024 mid January	Recurrence: right pleural dissemination	METex14 skipping positive, PD‐L1 10%
2024 late February	Tepotinib 500 mg daily	
2024 early May	Discontinued after 69 days due to ILD; improved with steroids	
2024 early July	CBDCA + pemetrexed ×3 cycles → pembrolizumab added	Temporary response; later progression with pleural effusion
2024 late September	Disease progression	Effusion, tumor growth
2024 early October	Capmatinib started	Discontinued after 10 days due to fever
2024 late October	Docetaxel + ramucirumab	Six cycles given
2025 late March	Gumarontinib started	Discontinued after 36 days due to hepatotoxicity
2025 late April	Gumarontinib restarted (150 mg/day)	Discontinued after 57 days due to edema and renal impairment
2025 late August—present	CBDCA + pemetrexed + bevacizumab	Ongoing chemotherapy regimen

## 3 Discussion

Gumarontinib, a selective MET tyrosine kinase inhibitor (TKI) developed in China, is the third MET‐TKI approved in Japan for the treatment of NSCLC harboring MET exon 14 skipping alterations. Alongside tepotinib and capmatinib, it is considered one of the key targeted therapies for MET exon 14 altered NSCLC.

Although MET‐TKIs demonstrate substantial therapeutic efficacy, treatment discontinuation due to adverse events remains a significant clinical challenge. ILD is particularly notable as a severe adverse event, with reported incidence rates of 6.1% in the VISION trial for tepotinib and 4.5% in the GEOMETRY mono‐1 trial for capmatinib. Among Japanese patients, the ILD incidence rose to 8.3% [1, 2]. In contrast, gumarontinib was primarily associated with hepatotoxicity, and the ILD incidence was reported at 1.2% [7]. While direct comparison is difficult due to differences in patient backgrounds, observation periods, and evaluation criteria across trials, these data offer useful guidance for clinical decision‐making. Table 2 summarizes these outcomes. Notably, ILD was not the most frequent cause of treatment discontinuation.

**TABLE 2:** Reported rates of adverse events and treatment discontinuation in pivotal trials of MET‐TKIs.

	Capmatinib	Tepotinib	Gumarontinib
Major adverse events	Edema, dysgeusia, liver dysfunction	Edema, liver dysfunction, loss of appetite	Liver dysfunction, fever
Reported frequency of ILD	2%–3%	6%	1.2%
Incidence of edema	51%	70%	10%
Incidence of liver dysfunction	20%	26%	30%
Mucosal and taste disorders	Frequent	Rare	Rare
Discontinuation rate	10%	7%–10%	5%–10%

*Note:* Data from VISION (tepotinib), GEOMETRY mono‐1(capmatinib), and a Chinese Phase II trial (gumarontinib) illustrate variability in toxicity profiles among agents.

In pivotal trials, the median overall survival was 17.1 months with tepotinib and 21.5 months with capmatinib [1, 2].

Recent case reports have demonstrated the potential efficacy of MET‐TKI rechallenge after resolution of ILD [3, 4, 5, 6]. This approach holds clinical value as a strategy to overcome resistance or for patients with limited therapeutic options. In the context of EGFR‐TKIs, rechallenge with a different TKI following discontinuation due to adverse effects has been shown to improve overall survival [8], suggesting the potential utility of a similar strategy for MET‐TKIs. However, in clinical practice, non‐ILD adverse events—such as skin rash, fatigue, peripheral edema, and liver dysfunction—often lead to treatment discontinuation within 1–3 months, making long‐term rechallenge difficult. In this case, treatment with tepotinib was discontinued due to Grade 3 ILD, while both capmatinib and gumarontinib had to be discontinued shortly thereafter due to non‐ILD toxicities (fever, mucositis, fatigue, and hepatotoxicity).

Reports by Hasnibuchi et al. and Yasuda et al. also noted that while ILD recurrence was not observed, non‐ILD toxicities were the main barrier to sustained rechallenge [9, 10]. Among rechallenge cases, long‐term continuation was only achieved in the report by Hashiguchi et al. (approximately 180 days), with most other cases discontinued within 10–90 days (Table 3). Table 3 summarizes five MET‐TKI rechallenge cases, including ours. The full treatment history, including chemotherapy and immunotherapy, is shown in Table S3. Although higher PD‐L1 expression may be associated with longer response duration, NSCLCs with MET exon 14 skipping mutations tend to exhibit high PD‐L1 but low TMB, making them generally less responsive to ICIs [11].

**TABLE 3:** Comparison of five MET‐TKI rechallenge cases, including the present one, highlighting treatment durations, toxicities, and discontinuation causes.

Study (Author)	Treatment sequence TKI	Initial/second TKI toxicity	PDL1	Observation period	TKI duration (days)	Toxicities during rechallenge
Kashizaki et al. [3]	Investigationaldrug (MET‐TKI) Tepotinib Capmatinib	Maculopapular rash/same	1%–4%	24 months	11/8/30	Unknown
Kunimasa et al. [4]	Tepotinib Capmatinib	Peripheral edema Grade 3	60%	36 months	14/100	Unremarkable
Tseng et al. [5]	Capmatinib Investigational drug(MET‐TKI) Tepotinib	ILD Grade 3/tumor progression	Negative	5 months	30/14/30	None except tumor progression
Hashiguchi et al. [6]	Capmatinib Tepotinib	ILD Grade 2	90%	4 years 7 months	6/180	
Current case	Tepotinib Capmatinib Gumarontinib	ILD Grade 3/fever Grade 3	10%	20 months	60/10/36 + 57	Hepatotoxity Grade 2

The reproducibility of toxicity across MET‐TKIs may be attributed to their shared pharmacological properties. Tepotinib, capmatinib, and gumarontinib are all reversible, Type I ATP‐competitive inhibitors. Although structurally distinct from agents such as EGFR‐TKIs, they may share a class‐related toxicity profile [12]. However, adverse events are not universally repeated in every patient; notably, none of the five documented cases, including the present case, experienced ILD recurrence, suggesting that MET‐TKI rechallenge may be a viable option in carefully selected patients, although no definitive conclusions can be drawn from this limited sample. Interindividual differences in adverse reactions may be explained by multiple interacting factors, including tissue distribution of the drugs, plasma concentrations, metabolic pathways (e.g., CYP3A4, UGT1A1), immune response variability, and prior treatment‐induced tissue sensitization [13].

Previous use of ICIs or combination chemotherapy may alter immune and metabolic environments, potentially lowering the threshold for TKI‐related toxicity. Indeed, one report found that the incidence of grade ≥ 3 adverse events increased to 27% when MET‐TKIs were administered after ICIs [14, 15], underscoring the importance of risk assessment based on treatment history. In this case [15], although 6 months had passed between the last pembrolizumab dose and gumarontinib rechallenge, non‐ILD toxicities still emerged, suggesting that ICI‐induced effects may persist even after prolonged intervals.

To our knowledge, this is the first report describing gumarontinib treatment in a patient with prior discontinuation of both tepotinib and capmatinib (based on a PubMed search as of April 2025). Although these MET‐TKIs share similar pharmacologic mechanisms, the pattern of adverse events may differ, and non‐ILD toxicities appear to be the most significant limitation to continued treatment.

Rechallenge with MET‐TKIs, when guided by careful assessment of toxicity risks, may offer a viable strategy, especially for patients whose prior discontinuation was due to adverse effects. Although the treatment duration was limited in this case, sequential administration of multiple TKIs enabled cumulative disease control, offering clinically relevant insight into the balance between therapeutic benefit and toxicity risk.

Moving forward, accumulation of more clinical cases and prospective trials will be critical to evaluate the safety and efficacy of rechallenge strategies. Additionally, identifying predictive biomarkers for toxicity, elucidating the link with immune‐environmental changes, and developing risk stratification models that consider treatment history are essential next steps.

## 4 Conclusion

This case represents the first report of sequential use of all three MET‐TKIs currently approved in Japan and highlights both the challenges and the potential of MET‐TKI rechallenge therapy.

In this patient, treatment discontinuation was not due to recurrent ILD but rather to distinct non‐ILD toxicities associated with each agent.

These findings suggest that toxicity profiles vary among MET‐TKIs and that rechallenge may remain feasible with careful monitoring and individualized risk assessment.

Further accumulation of clinical data and identification of predictive biomarkers will be essential to define safe conditions for MET‐TKI rechallenge in the future.

## 5 Limitations

This report describes a single patient, which limits the generalizability of the findings. In addition, pharmacokinetic analyses and mechanistic biomarkers were not available, precluding definitive differences in toxicity among sequential MET‐TKIs.

## Author Contributions

A.N. contributed significantly to the clinical management of the patients, study conception, and manuscript writing. T.K., K.I., H.I., and J.U. are also involved in clinical practice. H.I. and S.K. contributed to pathological examination and case review. All authors reviewed the final draft of the manuscript and approved its submission.

## Funding

The authors have nothing to report.

## Ethics Statement

This study was conducted in accordance with the ethical standards of the institution.

## Consent

Written informed consent was obtained from the patient for participation and publication of this case report and any accompanying images. Ethics committee approval was not required for this single‐patient case report.

## Conflicts of Interest

The authors declare no conflicts of interest.

## Supporting information


**Table S3:** Comprehensive treatment histories of the reported cases, including MET‐TKIs, chemotherapy, and immune checkpoint inhibitors.

## Data Availability

The data that support the findings of this study are available on request from the corresponding author. The data are not publicly available due to privacy or ethical restrictions.
