# Supplementary material for: Challenges and Limitations of Sequential MET ‐ TKI Therapy in METex14 ‐ Positive NSCLC With a Focus on Non‐ ILD Toxicities: A Case Report
Source: Cancer Rep (Hoboken). 2026 Jan 16;9(1):e70458. doi: 10.1002/cnr2.70458 (PMC12809259; doi:10.1002/cnr2.70458)
Supplement: Supplementary file 1 — Table S3: Comprehensive treatment histories of the reported cases, including MET‐TKIs, chemotherapy, and immune checkpoint inhibitors. [file CNR2-9-e70458-s001.docx]

| **Study (Author)** | **Treatment Sequence** |
| --- | --- |
| Kashizaki et al. (4) | 1. Investigational drug (MET-TKI)  2. CBDCA＋Pem＋ipilimumab＋Nivolumab 3. Tepotinib 4. Pembrolizumab 5. Capmatinib |
| Kunimasa et al. (5) | 1. Pembrolizumab  2. CBDCA＋Pemetrexed＋Bevacizumab  3. DTX＋Ram 4. Tepotinib 5. Capmatinib |
| Tseng et al. (6) | 1. Capmatinib  2. Investigational drug (MET-TKI)  3.　Tepotinib |
| Hashiguchi et al. (7) | 1. Pembrolizumab  2. CBDCA＋Pemetrexed＋Bevacizumab  3. CBDCA＋nab-PTX 4. tegaful, gimeracil, and oteracil potassium  5. Capmatinib 　6. Tepotinib |
| Current  Case | 1. Tepotinib 2. CBDCA＋Pem＋Pembrolizumab  3. Capmatinib  4. DTX＋Ram 5. Gumarontinib 6. CBDCA＋Pem＋Bev |

Supplementary Table-3S
